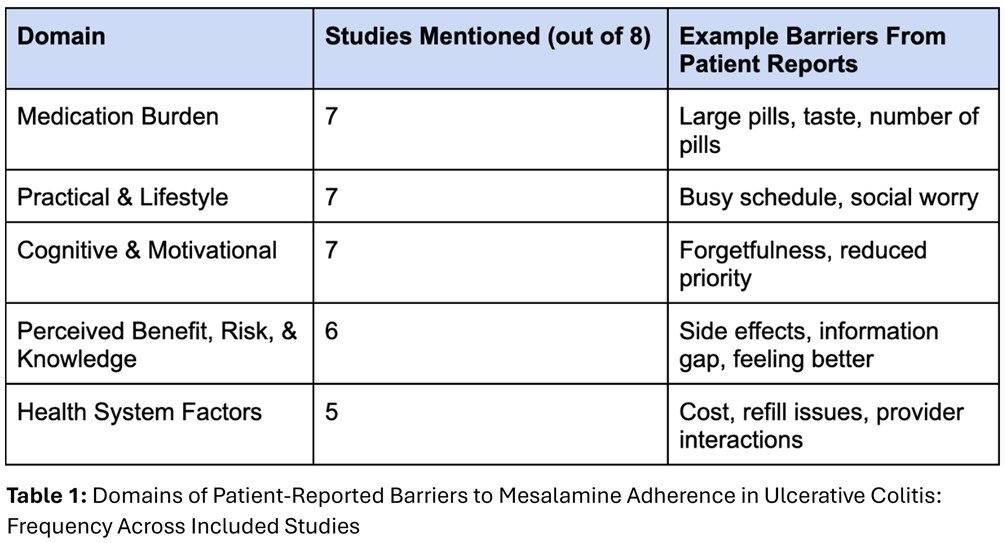# Poster Session II - A299 BARRIERS TO MESALAMINE ADHERENCE IN PATIENTS WITH MILD TO MODERATE ULCERATIVE COLITIS: A QUALITATIVE SYSTEMATIC REVIEW

**DOI:** 10.1093/jcag/gwaf042.299

**Published:** 2026-02-13

**Authors:** I Asaria, A Stolz, B Nguyen

**Affiliations:** The University of British Columbia Faculty of Medicine, Vancouver, BC, Canada; The University of British Columbia Faculty of Medicine, Vancouver, BC, Canada; The University of British Columbia Faculty of Medicine, Vancouver, BC, Canada

## Abstract

**Background:**

5-aminosalicylic acid (5-ASA), also known as mesalamine or mesalazine is the first-line treatment of choice for mild-moderate forms of ulcerative colitis (UC). Patients often prefer oral routes of administration compared to topical routes such as enema or suppository. However, differences in adherence to tablet and granular forms of mesalamine remain unclear. A clear understanding of the comparative adherence to mesalamine tablet and granular formulations is needed to guide optimal therapy and improve real-world management of UC.

**Aims:**

To describe reported reasons and factors for non-adherence when taking prescribed 5-ASA tablets and granules in the treatment of mild-moderate UC.

**Methods:**

We performed a qualitative systematic review according to PRISMA guidelines, searching Embase, MEDLINE, Web of Science, Scopus, and Pubmed. Data extraction consisted of standardized tables including study design, patient age, clinical features, treatment choice (either granules or tablets), self-reported adherence data, and qualitative data via interviews or questionnaires.

**Results:**

A total of 8 articles (4 focused interviews, 3 surveys, 1 discrete choice experiment; n = 848 mild-moderate UC patients) from 2008-2023 were included. Only 1 study reported qualitative data on patients using both granule and tablet formulations of mesalamine, while 7 focused solely on tablets. Adherence rates were not reported in all studies. Qualitative findings from each study were extracted and synthesized into five domains: 1) medication burden, 2) practical and lifestyle fit, 3) cognitive and motivational barriers, 4) perceived benefit, risk, & knowledge, and 5) health system factors (Table 1). The most commonly reported barriers were forgetfulness, followed by pill characteristics (dosing frequency and size) and perceived social implications.

**Conclusions:**

The established criteria for complete adherence to mesalamine among the included studies was patients taking 80% or more of their prescribed doses within the specified study period. Future interventions must consider addressing the domains found in this review including simplifying dosing regimens, enhancing patient education, tailoring support for integrating medication into daily routines, and improving healthcare access to optimize mesalamine adherence.

**Funding Agencies:**

None